# Measurement properties of translated versions of neck-specific questionnaires: a systematic review

**DOI:** 10.1186/1471-2288-11-87

**Published:** 2011-06-06

**Authors:** Jasper M Schellingerhout, Martijn W Heymans, Arianne P Verhagen, Henrica C de Vet, Bart W Koes, Caroline B Terwee

**Affiliations:** 1 Department of General Practice, Erasmus Medical Centre, Rotterdam, The Netherlands; 2 Department of Epidemiology and Biostatistics, EMGO Institute for Health and Care Research, VU University Medical Centre, Amsterdam, The Netherlands; 3 Department of Methodology and Applied Biostatistics, VU University Medical Centre, Amsterdam, The Netherlands

## Abstract

**Background:**

Several disease-specific questionnaires to measure pain and disability in patients with neck pain have been translated. However, a simple translation of the original version doesn't guarantee similar measurement properties. The objective of this study is to critically appraise the quality of the translation process, cross-cultural validation and the measurement properties of translated versions of neck-specific questionnaires.

**Methods:**

Bibliographic databases were searched for articles concerning the translation or evaluation of the measurement properties of a translated version of a neck-specific questionnaire. The methodological quality of the selected studies and the results of the measurement properties were critically appraised and rated using the COSMIN checklist and criteria for measurement properties.

**Results:**

The search strategy resulted in a total of 3641 unique hits, of which 27 articles, evaluating 6 different questionnaires in 15 different languages, were included in this study. Generally the methodological quality of the translation process is poor and none of the included studies performed a cross-cultural adaptation. A substantial amount of information regarding the measurement properties of translated versions of the different neck-specific questionnaires is lacking. Moreover, the evidence for the quality of measurement properties of the translated versions is mostly limited or assessed in studies of poor methodological quality.

**Conclusions:**

Until results from high quality studies are available, we advise to use the Catalan, Dutch, English, Iranian, Korean, Spanish and Turkish version of the NDI, the Chinese version of the NPQ, and the Finnish, German and Italian version of the NPDS. The Greek NDI needs cross-cultural validation and there is no methodologically sound information for the Swedish NDI. For all other languages we advise to translate the original version of the NDI.

## Background

Several disease-specific questionnaires have been developed to measure pain and disability in patients with neck pain (e.g. Neck Disability Index (NDI), Neck Pain and Disability Scale (NPDS)) [1,2]. To make them suitable for use in other languages, several of these neck-specific questionnaires have been translated. However, a simple translation of the original version doesn't guarantee similar measurement properties, because differences in cultural context have to be taken into account as well [3,4].

Previous reviews of neck-specific questionnaires have not paid sufficient attention to possible differences in performance, caused by differences in cultural context, and combine the results of studies that evaluate measurement properties of different language versions of the same questionnaire [5,6]. This may lead to inconsistent results for measurement properties, as was demonstrated in a recent review of the cross-cultural adaptations of the McGill Pain Questionnaire [7].

Since it is possible that the measurement properties of neck-specific questionnaires vary between different nationalities, we decided to evaluate them per language. This reduces inconsistency in results due to cultural differences and also facilitates a choice for the best questionnaire per language. The measurement properties of original versions of the different neck-specific questionnaires were evaluated in a separate systematic review. (Schellingerhout JM, Heymans MW, Verhagen AP, De Vet HC, Koes BW, Terwee CB: Measurement properties of disease-specific questionnaires in patients with neck pain: a systematic review, submitted)

The purpose of this study is to critically appraise the quality of the translation process, cross-cultural validation and the measurement properties of translated versions of neck-specific questionnaires.

## Methods

### Search strategy

We searched the following computerised bibliographic databases: Medline (1966 to July 2010), EMbase (1974 to July 2010), CINAHL (1981 to July 2010), and PsycINFO (1806 to July 2010). We used the index terms "neck", "neck pain", and "neck injuries/injury" in combination with "research measurement", "questionnaire", "outcome assessment", "psychometry", "reliability", "validity", and derivatives of these terms. The full search strategy used in each database is available upon request from the corresponding author. Reference lists were screened to identify additional relevant studies.

### Selection criteria

A study was included if it was a full text original article (e.g. not an abstract, review or editorial), published in English, concerning the translation or evaluation of the measurement properties of a translated version of a neck-specific questionnaire. The questionnaire had to be self-reported, evaluating pain and/or disability, and specifically developed or adapted for patients with neck pain.

For inclusion, neck pain had to be the main complaint of the study population. Accompanying complaints (e.g. low back pain or shoulder pain) were no reason for exclusion, as long as the main focus was neck pain. Studies considering study populations with a specific neck disorder (e.g. neurological disorder, rheumatological disorder, malignancy, infection, or fracture) were excluded, except for patients with cervical radiculopathy or whiplash associated disorder (WAD).

Two reviewers (JMS, APV) independently assessed the titles, abstracts, and reference lists of studies retrieved by the literature search. In case of disagreement between the two reviewers, there was discussion to reach consensus. If necessary, a third reviewer (HCV) made the decision regarding inclusion of the article.

### Measurement properties

The measurement properties are divided over three domains: reliability, validity, and responsiveness [8]. In addition, the interpretability is described.

#### Reliability

Reliability is defined as the extent to which scores for patients who have not changed are the same for repeated measurement under several conditions: e.g. using different sets of items from the same questionnaire (internal consistency); over time (test-retest); by different persons on the same occasion (inter-rater); or by the same persons on different occasions (intra-rater) [8].

Reliability contains the following measurement properties:

- *Internal consistency: *The interrelatedness among the items in a questionnaire, expressed by Cronbach's α or Kuder-Richardson Formula 20 (KR-20) [8,9].

- *Measurement error: *The systematic and random error of a patient's score that is not attributed to true changes in the construct to be measured, expressed by the standard error of measurement (SEM) [8,10]. The SEM can be converted into the smallest detectable change (SDC) [10]. Changes exceeding the SDC can be labeled as change beyond measurement error [10]. Another approach is to calculate the limits of agreement (LoA) [11]. For determining the adequacy of measurement error the SDC and/or LoA is related to the minimal important change (MIC) [12].

- *Reliability: *The proportion of the total variance in the measurements which is due to 'true' differences between patients [8]. This aspect is reflected by the Intraclass Correlation Coefficient (ICC) or Cohen's Kappa [8,13].

#### Validity

Validity is the extent to which a questionnaire measures the construct it is supposed to measure and contains the following measurement properties [8]:

- *Content validity: *The degree to which the content of a questionnaire is an adequate reflection of the construct to be measured [8]. Important aspects are whether all items are relevant for the construct, aim, and target population and if no important items are missing (comprehensiveness) [14].

- *Criterion validity: *The extent to which scores on an instrument are an adequate reflection of a gold standard [8]. Since a real gold standard for health status questionnaires is not available, [14] we will not evaluate criterion validity.

- *Construct validity *is divided into three aspects:

• *Cross-cultural validity: *The degree to which the performance of the items on a translated or culturally adapted instrument are an adequate reflection of the performance of the items of the original version of the instrument [8]. This is assessed by means of multi-group factor analysis or differential item functioning using data from a population that completed the questionnaire in the original language, as well as data from a population that completed the questionnaire in the new language.

• *Structural validity: *The degree to which the scores of an instrument are an adequate reflection of the dimensionality of the construct to be measured [8]. Factor analysis should be performed to confirm the number of subscales present in a questionnaire [14].

• *Hypothesis testing: *The degree to which a particular measure relates to other measures in a way one would expect if it is validly measuring the supposed construct, i.e. in accordance with predefined hypotheses about the correlation or differences between the measures [8].

#### Responsiveness

Responsiveness is the ability of an instrument to detect change over time in the construct to be measured [8]. Responsiveness is considered an aspect of validity, in a longitudinal context [14]. Therefore, the same standards apply as for validity: the correlation between change scores of two measures should be in accordance with predefined hypotheses [14]. Another approach is to consider the measurement instrument as a diagnostic test to distinguish improved and non-improved patients. The responsiveness of the instrument is then expressed as the area under the receiver operator characteristic curve (AUC) [14].

#### Interpretability

Interpretability is the degree to which one can assign qualitative meaning to quantitative scores [8]. This means that investigators should provide information about clinically meaningful differences in scores between subgroups, floor and ceiling effects, and the MIC [14]. Interpretability is not a measurement property, but an important characteristic of a measurement instrument [8].

### Quality assessment

Assessment of the methodological quality of the selected studies was carried out using the COSMIN checklist [9]. The COSMIN checklist consists of nine boxes with methodological standards for how each measurement property should be assessed. Each item was scored on a 4-point rating scale (i.e. "poor", "fair", "good", or "excellent", see http://www.cosmin.nl). An overall score for the methodological quality of a study was determined by taking the lowest rating of any of the items in a box. The methodological quality of a study was evaluated per measurement property. Special attention was paid to the methodological quality of the translation process and cross-cultural validation. The COSMIN box concerning this measurement property is presented in Table 1.

**Table 1:** Methodological criteria for the translation process and cross-cultural validation [9]

Item	Methodological Criteria
1	Was the percentage of missing items given?
2	Was there a description of how missing items were handled?
3	Was the sample size included in the analysis adequate?
4	Were both the original language in which the HR-PRO instrument was developed,
	and the language in which the HR-PRO instrument was translated described?
5	Was the expertise of the people involved in the translation process adequately described?
	e.g. expertise in the disease(s) involved, in the construct to be measured, or in both languages
6	Did the translators work independently from each other?
7	Were items translated forward and backward?
8	Was there an adequate description of how differences between the original and
	translated versions were resolved?
9	Was the translation reviewed by a committee (e.g. original developers)?
10	Was the HR-PRO instrument pre-tested (e.g. cognitive interviews) to check interpretation,
	cultural relevance of the translation, and ease of comprehension?
11	Was the sample used in the pre-test adequately described?
12	Were the samples similar for all characteristics except language and/or cultural background?
13	Were there any important flaws in the design or methods of the study?
14	for CTT: Was confirmatory factor analysis performed?
15	for IRT: Was differential item function (DIF) between language groups assessed?

CTT = Classical Test Theory, IRT = Item Response Theory

Data extraction and assessment of (methodological) quality were performed by two reviewers (JMS, CBT) independently. In case of disagreement between the two reviewers, there was discussion in order to reach consensus. If necessary, a third reviewer (HCV) made the decision.

### Best evidence synthesis - levels of evidence

To determine the overall quality of the measurement properties of the different questionnaires we synthesized the different studies per language by combining their results, adjusted for methodological quality of the studies and the consistency of their results. The possible overall rating for a measurement property is "positive", "indeterminate", or "negative", accompanied by levels of evidence, similarly as was proposed by the Cochrane Back Review Group (see Table 2) [15,16].

**Table 2:** Levels of evidence for the overall quality of the measurement property [16]

Level	Rating	Criteria
strong	+++ or ---	Consistent findings in multiple studies of good
		methodological quality OR in one study of excellent
		methodological quality
moderate	++ or --	Consistent findings in multiple studies of fair
		methodological quality OR in one study of good
		methodological quality
limited	+ or -	One study of fair methodological quality
conflicting	+/-	Conflicting findings
unknown	?	Only studies of poor methodological quality

[..] = reference number

+ = positive result, - = negative result

To assess whether the results of the measurement properties were positive, negative, or indeterminate, we used criteria based on Terwee et al. (see Table 3) [17].

**Table 3:** Quality criteria for measurement properties [Based on Terwee et al., [17]]

Property	Rating	Quality Criteria
**Reliability**		
Internal consistency	+	(Sub)scale unidimensional AND Cronbach's alpha(s) ≥ 0.70
	?	Dimensionality not known OR Cronbach's alpha not determined
	-	(Sub)scale not unidimensional OR Cronbach's alpha(s) < 0.70
Measurement error	+	MIC > SDC OR MIC outside the LOA
	?	MIC not defined
	-	MIC ≤ SDC OR MIC equals or inside LOA
Reliability	+	ICC/weighted Kappa ≥ 0.70 OR Pearson's r ≥ 0.80
	?	Neither ICC/weighted Kappa, nor Pearson's r determined
	-	ICC/weighted Kappa < 0.70 OR Pearson's r < 0.80
**Validity**		
Content validity	+	The target population considers all items in the questionnaire to be relevant
		AND considers the questionnaire to be complete
	?	No target population involvement
	-	The target population considers items in the questionnaire to be irrelevant
		OR considers the questionnaire to be incomplete
Construct validity		
- Cross-cultural validity	+	Original factor structure confirmed OR no important DIF
	?	Confirmation original factor structure AND DIF not mentioned
	-	Original factor structure not confirmed OR important DIF
- Structural validity	+	Factors should explain at least 50% of the variance
	?	Explained variance not mentioned
	-	Factors explain < 50% of the variance
- Hypothesis testing	+	(Correlation with an instrument measuring the same construct ≥ 0.50 OR
		at least 75% of the results are in accordance with the hypotheses) AND
		correlation with related constructs is higher than with unrelated constructs
	?	Solely correlations determined with unrelated constructs
	-	Correlation with an instrument measuring the same construct < 0.50 OR
		< 75% of the results are in accordance with the hypotheses OR
		correlation with related constructs is lower than with unrelated constructs
**Responsiveness**		
Responsiveness	+	(Correlation with an instrument measuring the same construct ≥ 0.50
		OR at least 75% of the results are in accordance with the hypotheses
		OR AUC ≥ 0.70) AND correlation with related constructs is higher
		than with unrelated constructs
	?	Solely correlations determined with unrelated constructs
	-	Correlation with an instrument measuring the same construct < 0.50 OR
		< 75% of the results are in accordance with the hypotheses OR AUC < 0.70
		OR correlation with related constructs is lower than with unrelated constructs

[..] = reference number, MIC = minimal important change, SDC = smallest detectable change, LOA = limits of agreement, ICC = intraclass correlation coefficient, DIF = differential item functioning, AUC = area under the curve

^† ^+ = positive rating, ? = indeterminate rating, - = negative rating

## Results

**Figure 1:**
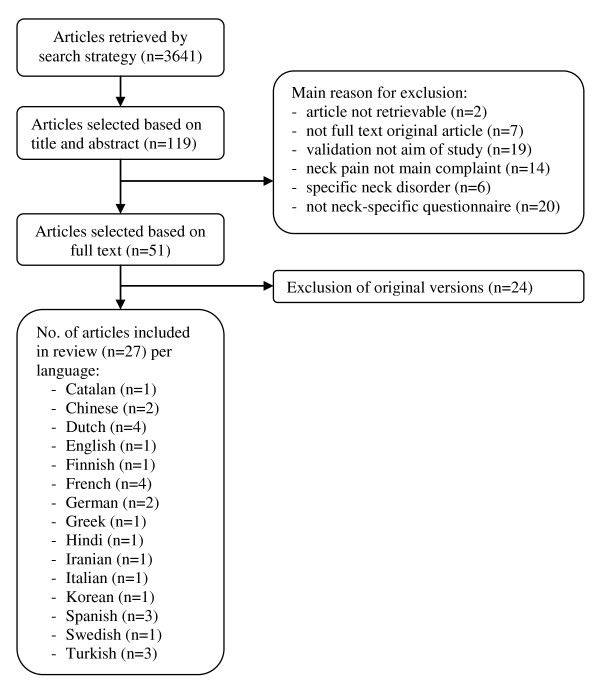
**Flowchart search and selection**.

The search strategy resulted in a total of 3641 unique hits, of which 119 articles were selected based on their title and abstract. The full text assessment resulted in exclusion of another 68 articles. Reference checking did not result in additional articles. Twenty-four articles concerned original versions of neck-specific questionnaires, which were evaluated in a separate systematic review. (Schellingerhout JM, Heymans MW, Verhagen AP, De Vet HC, Koes BW, Terwee CB: Measurement properties of disease-specific questionnaires in patients with neck pain: a systematic review, submitted) Finally, 27 articles on translated questionnaires, evaluating 6 different questionnaires in 15 different languages, were included in this study (see Figure 1).

The general characteristics of these studies are presented in Table 4. None of the included studies performed a cross-cultural validation (Table 1, items 14 and 15), i.e. no studies performed multi-group factor analysis or differential item functioning. Therefore, we were only able to rate the methodological quality of the translation process (Table 1, items 4-11). The methodological quality of the studies is presented in Table 5 for each measurement property, arranged per language. Generally the methodological quality of the studies was poor to fair. The synthesis of the results per questionnaire and their accompanying level of evidence is presented in Table 6 for each language. For each questionnaire, except for the Iranian NPDS and Spanish NDI, at least half of the information regarding measurement properties is lacking. Moreover, the evidence for the quality of measurement properties is mostly limited, due to methodological shortcomings of the included studies.

**Table 4:** General information per study

Study	Language	Country	Population	Setting
Nieto et al. [25]	Catalan	Spain	< 3 months whiplash	rehabilitation unit
Chiu et al. [26]	Chinese	Hong Kong	neck pain	physiotherapist
Lee et al. [27]	Chinese	Hong Kong	neck pain	physiotherapist
Jorritsma et al. [19]	Dutch	Netherlands	> 3 months non-specific neck pain	rehabilitation unit
Pool et al. [29]	Dutch	Netherlands	non-specific neck pain	general practitioner
Schmitt et al. [30]	Dutch	Netherlands	> 3 weeks whiplash	general population
Vos et al. [31]	Dutch	Netherlands	< 6 weeks non-specific neck pain	general practitioner
Stewart et al. [33]	English	Australia	> 3 months whiplash	physiotherapist
Salo et al. [35]	Finnish	Finland	neck pain	physiotherapist/rehabilitation unit
Forestier et al. [18]	French	France	> 3 months mechanical neck pain	general population
Martel et al. [37]	French	Canada	> 12 weeks mechanical neck pain	general population
Wlodyka-Demaille et al. [36]	French	France	> 15 days non-specific neck pain	rehabilitation unit/rheumatologist
Wlodyka-Demaille et al. [20]	French	France	> 15 days non-specific neck pain	rehabilitation unit/rheumatologist
Bremerich et al. [24]	German	Switzerland	> 3 months non-specific neck pain	rheumatologist
Scherer et al. [38]	German	Germany	neck pain	general practitioner
Trouli et al. [39]	Greek	Greece	non-specific neck pain	primary care
Agarwal et al. [40]	Hindi	India	cervical radiculopathy	physiotherapist
Mousavi et al. [41]	Iranian	Iran	non-specific neck pain	primary care/physiotherapist
Monticone et al. [42]	Italian	Italy	> 4 weeks non-specific neck pain	rehabilitation unit
Lee et al. [43]	Korean	South Korea	non-specific neck pain	physiotherapist
Andrade et al. [46]	Spanish	Spain	non-specific neck pain	rehabilitation unit
Gonzalez et al. [44]	Spanish	Spain	> 4 months non-specific neck pain	physiotherapist
Kovacs et al. [23]	Spanish	Spain	non-specific neck pain	primary care/hospital outpatient clinic
Ackelman et al. [22]	Swedish	Sweden	acute/chronic neck pain	emergency room/physiotherapist
Aslan et al. [47]	Turkish	Turkey	> 3 months non-specific neck pain	physiotherapist/rehabilitation unit
Bicer et al. [21]	Turkish	Turkey	> 6 months non-specific neck pain	rehabilitation unit
Kose et al. [48]	Turkish	Turkey	> 6 weeks non-specific neck pain	primary care

[..] = reference number

**Table 5:** Methodological quality of each study per measurement property

Language		Translation	Internal	Measurement		Content	Structural	Hypotheses	
Study	Instrument	process	Consistency	Error	Reliability	Validity	Validity	Testing	Responsiveness
**Catalan**									
Nieto et al. [25]	NDI	poor	good				fair	good	
**Chinese**									
Chiu et al. [26]	NPQ	poor	poor		excellent	poor		fair	poor
Lee et al. [27]	NPQ							fair	poor
**Dutch**									
Jorritsma et al. [19]	NDI			poor	poor				
	NPDS	fair		poor	poor				
Pool et al. [29]	NDI			fair					fair
Schmitt et al. [30]	NBQ	excellent	poor	fair	fair			poor	
Vos et al. [31]	NDI			fair	fair				poor
**English**									
Stewart et al. [33]	CNFDS								fair
**Finnish**									
Salo et al. [35]	NDI	poor	excellent		poor		good	poor	
	NPDS	poor	excellent		poor		good	poor	
**French**									
Forestier et al. [18]	CNFDS	poor	poor						poor
Martel et al. [37]	NBQ	poor			poor			fair	moderate
Wlodyka et al. [36]	NDI	poor	poor	poor	poor		fair	fair	
	NPDS	poor	poor	poor	poor		fair	fair	
	NPQ	poor	poor	poor	poor		fair	fair	
Wlodyka et al. [20]	NDI								poor
	NPDS								poor
	NPQ								poor
**German**									
Bremerich et al. [24]	NPDS	fair		poor	poor				
Scherer et al. [38]	NPDS	poor	excellent				good	good	
**Greek**									
Trouli et al. [39]	NDI	good	good	poor	poor		good		fair
**Hindi**									
Agarwal et al. [40]	NPDS	fair	poor	poor	poor	poor		fair	
**Iranian**									
Mousavi et al. [41]	NDI	excellent	fair		fair	poor			fair
	NPDS	excellent	fair		fair	poor	fair		fair
**Italian**									
Monticone et al. [42]	NPDS	poor	fair		fair		fair	poor	
**Korean**									
Lee et al. [43]	NDI	poor	fair	poor	poor			fair	poor
	NPDS	poor	poor	poor	poor			fair	poor
**Spanish**									
Andrade et al. [46]	NDI		fair	poor	poor		fair	fair	fair
Gonzalez et al. [44]	NPQ	poor	poor		fair			poor	poor
Kovacs et al. [23]	NDI	excellent	poor		poor			poor	poor
	NPQ		poor					poor	poor
	CNQ	excellent	poor		poor			poor	poor
**Swedish**									
Ackelman et al. [22]	NDI				poor	poor		poor	
**Turkish**									
Aslan et al. [47]	NDI	excellent			fair			fair	
Bicer et al. [21]	NPDS	poor	poor					poor	
Kose et al. [48]	NDI	fair	poor		fair			poor	fair
	NPDS	fair	poor		fair			poor	fair
	NPQ	fair	poor		fair			poor	fair
	CNFDS	fair	poor		fair			poor	fair

[..] = reference number

**Table 6:** Quality of the measurement properties per language and questionnaire

		Internal	Measurement		Content	Structural				Hypotheses	
Language	Instrument	Consistency	Error	Reliability	Validity	**Validity**^†^				Testing	Responsiveness
						1	2	3	4		
**Catalan**	NDI	++	na	na	na	-	+			++	na
**Chinese**	NPQ	?	na	+++	?	na				++	?
**Dutch**	NDI	na	-	+	na	na				na	+
	NPDS	na	?	?	na	na				na	na
	NBQ	?	?	+	na	na				?	na
**English**	CNFDS	na	na	na	na	na				na	+
**Finnish**	NDI	?	na	?	na	--				?	na
	NPDS	+++	na	?	na			++		?	na
**French**	NDI	na	?	?	na		+			-	?
	NPDS	na	?	?	na			+		+/-	?
	NBQ	na	na	?	na	na				+/-	-
	NPQ	na	?	?	na		+			+/-	?
	CNFDS	?	na	na	na	na				na	?
**German**	NPDS	?	?	?	na	--		++		++	na
**Greek**	NDI	?	?	?	na	--				na	-
**Hindi**	NPDS	?	?	?	?	na				+/-	na
**Iranian**	NDI	+	na	+	?	na				na	+
	NPDS	+	na	+	?				+	na	-
**Italian**	NPDS	+	na	+	na			+		?	na
**Korean**	NDI	+	?	?	na	na				?	?
	NPDS	?	?	?	na	na				?	?
**Spanish**	NDI	+	na	?	na	+				+	+
	NPQ	?	na	-	na	na				?	?
	CNQ	?	na	?	na	na				?	?
**Swedish**	NDI	na	na	?	?	na				?	na
**Turkish**	NDI	?	na	++	na	na				+	+
	NPDS	?	na	+	na	na				?	+
	NPQ	?	na	+	na	na				?	+
	CNFDS	?	na	+	na	na				?	+

+++ or --- = strong evidence positive/negative result, ++ or -- = moderate evidence positive/negative result, + or - = limited evidence positive/negative result, +/- = conflicting evidence, ? = unknown, due to poor methodological quality, na = no information available

^† ^the numbers reflect the number of factors that are mentioned in the underlying studies

Below we will discuss the results for the different questionnaires per language. The results regarding measurement properties from studies of poor methodological quality are not mentioned [18-24].

### Catalan

The NDI is the only neck-specific questionnaire that has been translated in Catalan [25]. The NDI was originally designed to measure activities of daily living (ADL) in patients with neck pain [1]. The methodological quality of the translation process is poor [25]. Confirmatory factor analysis showed that the NDI is not unidimensional and there is limited evidence that the NDI has a 2-factor structure [25]. Assuming a 2-factor structure, there is moderate positive evidence for internal consistency: Cronbach's α is 0.70 for "pain and interference with cognitive functioning" and 0.83 for "functional disability" [25]. There is a positive correlation (r = 0.51) between the NDI and the Pain Intensity Index [25].

The available evidence on measurement properties of the Catalan NDI is positive, despite the poor methodological quality of the translation process.

### Chinese

The Northwick Park Neck Pain Questionnaire (NPQ) is the only neck-specific questionnaire that has been translated in Chinese [26-28]. The NPQ was originally designed to measure the influence of non-specific neck pain on daily activities [29]. The methodological quality of the translation process is poor [26].

There is strong positive evidence for the reliability of the NPQ (ICC = 0.95) [26]. Hypothesis testing resulted in moderate positive evidence for correlation between the NPQ and instruments measuring pain and physical functioning (r = 0.59-0.75) [26,27]. Differences in score between subgroups have been reported (e.g. healthy persons vs. neck pain patients, and patients who sought medical consultation vs. those who did not) [26]. The average time needed to fill out the NPQ is 5.5 minutes [26].

The available information on measurement properties of the Chinese NPQ looks promising, despite the poor methodological quality of the translation process.

### Dutch

The NDI, NPDS, and Neck Bournemouth Questionnaire (NBQ) have been translated in Dutch [19,29-31]. The NPDS was originally designed to measure pain and disability in patients with neck pain [2]. The NBQ was originally designed to measure pain, physical functioning, social functioning, and psychological functioning in patients with non-specific neck pain [32]. The translation process of the NDI is not described, so the quality of this process is unknown. The methodological quality of the translation process of the NDPS is fair, [19] and of the NBQ is excellent [30].

There is limited positive evidence for the reliability of the NDI (ICC = 0.90), [31] and for responsiveness (sensitivity = 0.9 and specificity = 0.7 for a clinically important change of 3.5) [29]. There is limited negative evidence for its measurement error (MIC = 3.5 and SDC = 10.5 on a 0-50 scale) [29]. There is limited positive evidence for the reliability of the NBQ (ICC = 0.92) [30]. The result for measurement error of the NBQ is indeterminate, because the MIC is not defined [30]. No floor or ceiling effects have been detected for the NDI or NBQ, and for both questionnaires differences in score between subgroups have been reported (men vs. women) [30,31].

The lack of information derived from these studies makes it difficult to point out the best available neck-specific questionnaire in Dutch. Based on the information available on the measurement properties of the original version of the NDI and NBQ, we advise to use the Dutch NDI. (Schellingerhout JM, Heymans MW, Verhagen AP, De Vet HC, Koes BW, Terwee CB: Measurement properties of disease-specific questionnaires in patients with neck pain: a systematic review, submitted)

### English

The, originally Danish, Copenhagen Neck Functional Disability Scale (CNFDS) is the only neck-specific questionnaire that has been translated in English [33]. The CNFDS was originally designed to measure disability in patients with neck pain [34]. The translation process is not described, so the quality of this process is unknown. There is limited positive evidence for the responsiveness of the CNFDS (AUC = 0.73) [33]. Many neck-specific questionnaires have originally been developed in English. We advise to use one of these questionnaires, preferably the NDI. (Schellingerhout JM, Heymans MW, Verhagen AP, De Vet HC, Koes BW, Terwee CB: Measurement properties of disease-specific questionnaires in patients with neck pain: a systematic review, submitted)

### Finnish

The NDI and NPDS have been translated in Finnish [35]. The methodological quality of the translation process of these questionnaires is poor [35].

There is moderate evidence that the NDI is not one-dimensional and that the NPDS has a 3-factor structure [35]. The result for internal consistency of the NDI is indeterminate, because the authors unjustly assume a 1-factor model [35]. There is strong positive evidence for the internal consistency of the NPDS (Cronbach α = 0.82-0.84) [35]. No floor or ceiling effects have been detected for the NDI or NPDS and for both questionnaires differences in score between subgroups have been reported (stable vs. improved patients) [35].

The available information suggests that the Finnish NPDS has better measurement properties than the Finnish NDI.

### French

The following neck-specific questionnaires have been translated in French: NDI, [20,36] NPDS, [20,36] NBQ, [37] NPQ, [20,36] and CNFDS [18]. The methodological quality of all these translation processes is poor [18,36,37].

There is limited evidence that the NDI has a 2-factor structure [20]. Hypothesis testing showed that the correlation of the NDI with an instrument measuring psychological functioning is somewhat higher (r = 0.55), than with instruments measuring pain (r = 0.48), and physical functioning (r = 0.50) [20]. There is limited evidence that the NPDS has a 3-factor structure [20]. Hypothesis testing showed a positive result for correlation of the NPDS with instruments measuring pain (r = 0.52), and physical functioning (r = 0.63), and a negative result (results slightly below the pre-set criterion of r = 0.5) for correlation with instruments measuring psychological functioning (r = 0.40-0.49) [20]. Hypothesis testing showed a positive result for correlation of the NBQ with an instrument measuring pain and physical functioning (r = 0.61-0.67), and a negative result for correlation with an instrument measuring psychological functioning (r = 0.17-0.25) [37]. There is limited negative evidence for the responsiveness of the NBQ (r = 0.42) [37]. There is limited evidence that the NPQ has a 2-factor structure [20]. Hypothesis testing showed a positive result for correlation of the NPQ with an instrument measuring physical functioning (r = 0.53), and a negative result for correlation with an instrument measuring pain (r = 0.43) [20].

No floor or ceiling effects have been detected for the NDI, NPDS, and NPQ [20,36]. The average time needed to fill out the NDI, NPDS, and NPQ is 7.4, 6.4, and 7.2 minutes, respectively [36].

The lack of information derived from these studies makes it difficult to point out the best available neck-specific questionnaire in French. Based on the information available on the measurement properties of the original version of the NDI, NPDS, NBQ, NPQ, and CNFDS, we advise to develop a high quality translation of the NDI. (Schellingerhout JM, Heymans MW, Verhagen AP, De Vet HC, Koes BW, Terwee CB: Measurement properties of disease-specific questionnaires in patients with neck pain: a systematic review, submitted)

### German

The NPDS is the only neck-specific questionnaire that has been translated in German [24,38]. There are two translations of the NPDS in German: one translation process of poor and one of fair methodological quality [24,38].

Factor analysis provided moderate evidence that the NPDS has a 3-factor structure [38]. The result for internal consistency is indeterminate, [38] because the authors unjustly assume a 1-factor model. There is moderate positive evidence for hypothesis testing (>75% of results in accordance with predefined hypotheses) [38]. No floor or ceiling effects have been detected for the NPDS [38].

The available information on measurement properties of the German NPDS looks promising, despite the poor methodological quality of the translation process.

### Greek

The NDI is the only neck-specific questionnaire that has been translated in Greek [39]. The methodological quality of the translation process is good [39].

Exploratory factor analysis provided moderate evidence that the NDI does not have a 1-factor structure [39]. The result for internal consistency is indeterminate, [39] because the authors unjustly assume a 1-factor model. There is limited negative evidence for responsiveness (r = 0.30 with Global Rating of Change) [39].

Based on the good quality of the translation process and the negative results for unidimensionality and responsiveness, we advise to perform a cross-cultural validation of the Greek NDI.

### Hindi

The NPDS is the only neck-specific questionnaire that has been translated in Hindi [40]. The methodological quality of the translation process is fair [40].

Hypothesis testing showed a positive result for correlation of the NPDS with an instrument measuring psychological functioning (r = 0.80), and a negative result for correlation with an instrument measuring pain (r = 0.30), and an instrument measuring physical functioning (r = 0.15). The average time needed to fill out the NPDS was 8 minutes [40].

Based on the information derived from this study, we advise to develop a high quality translation of the NDI.

### Iranian

The NDI and NPDS have been translated in Iranian [41]. The methodological quality of the translations processes is excellent [41].

There is limited positive evidence for the internal consistency (Cronbach alpha = 0.88, assuming a 1-factor structure), reliability (ICC = 0.97), and responsiveness (r = 0.65 for physical functioning and r = 0.70 for pain) of the NDI [41]. Exploratory factor analysis resulted in limited positive evidence for a 4-factor structure of the NPDS [41]. There is limited positive evidence for internal consistency (Cronbach alpha = 0.75-0.94 for the four subscales), and reliability (ICC = 0.97) [41]. There is limited negative evidence for responsiveness of the NPDS, because correlation with change scores on instruments measuring the same constructs was lower than correlation with instruments measuring other constructs [41]. No floor or ceiling effects have been detected for the NDI or NPDS [41].

The Iranian NDI and NPDS both seem to have adequate measurement properties, but we advise using the NDI, based on the negative result for responsiveness of the NPDS and the good measurement properties of the original version of the NDI. (Schellingerhout JM, Heymans MW, Verhagen AP, De Vet HC, Koes BW, Terwee CB: Measurement properties of disease-specific questionnaires in patients with neck pain: a systematic review, submitted)

### Italian

The NPDS is the only neck-specific questionnaire that has been translated in Italian [42]. The methodological quality of the translation process is poor [42].

There is limited evidence that the NPDS has a 3-factor structure (variance = 63%) [42]. A confirmatory analysis with 4 factors showed a small improvement in variance (67%) [42]. Assuming a 3-factor structure, there is limited positive evidence for internal consistency: Cronbach α was 0.92 for "neck dysfunction related to general activities", 0.86 for "cognitive-behavioral aspects", and 0.89 for "neck dysfunction related to activities of the cervical spine" [42]. There is limited positive evidence for the reliability of the NPDS (r = 0.89-0.93) [42]. The average time needed to fill out the NPDS is 7.5 minutes [42].

The available information on measurement properties of the Italian NPDS looks promising, despite the poor methodological quality of the translation.

### Korean

The NDI and NPDS have been translated in Korean [43]. The methodological quality of the translation processes is poor [43].

There is limited positive evidence regarding the internal consistency of the NDI (Cronbach α = 0.92, assuming a 1-factor structure) [43]. No floor or ceiling effects have been detected for the NDI or NPDS and differences in score between subgroups have been reported (neck pain patients vs. healthy persons) [43].

Lack of information makes it difficult to point out whether the Korean NDI or NPDS has the best measurement properties. Based on the information available on the measurement properties of the original version of the NDI and NPDS, we advise to use the Korean NDI. (Schellingerhout JM, Heymans MW, Verhagen AP, De Vet HC, Koes BW, Terwee CB: Measurement properties of disease-specific questionnaires in patients with neck pain: a systematic review, submitted)

### Spanish

The NDI, NPQ, and Core Neck Questionnaire (CNQ) have been translated in Spanish [23,44]. The CNQ was originally designed to measure outcomes of care in patients with non-specific neck pain [45]. The methodological quality of the translation process of the NPQ is poor, [44] and of the NDI and CNQ is excellent [23].

There is limited positive evidence for a 1-factor structure of the NDI and its internal consistency (Cronbach α = 0.89) [46]. Hypothesis testing showed a positive result for correlation of the NDI with an instrument measuring pain (r = 0.65), and an instrument measuring physical functioning (r = 0.89) [46]. There is limited positive evidence for the responsiveness of the NDI [46]. There is limited negative evidence regarding the reliability of the NPQ (ICC = 0.63) [44]. No floor or ceiling effects have been detected for the NDI, NPQ, or CNQ, and scores across different categories of pain intensity have been reported [23]. The average time needed to fill out the NDI and CNQ is 4.0 and 2.1 minutes, respectively [23].

Based on the available information, we advise to use the Spanish NDI.

### Swedish

The NDI is the only neck-specific questionnaire that has been translated in Swedish [22]. The methodological quality of the translation process is unknown. No floor or ceiling effects have been detected for the NDI [22].

Based on the lack of information, we advise to perform high quality studies to fill in the missing information on the measurement properties of the Swedish NDI.

### Turkish

The following neck-specific questionnaires have been translated and evaluated in Turkish: NDI, [47,48] NPDS, [21,48] NPQ, [48] and CNFDS [48]. There are two translations of the NDI in Turkish: one translation process was of excellent methodological quality, [47] and one of fair methodological quality [48]. There are two translations of the NPDS as well: one translation process was of poor methodological quality, [21] and one of fair methodological quality [48]. The translation processes of the NPQ and CNFDS are both of fair methodological quality [48].

There is moderate positive evidence for the reliability of the NDI (ICC = 0.86-0.98), [47,48] and limited positive evidence for hypothesis testing (r = 0.66-0.73 with instruments measuring pain and/or disability) and responsiveness (r = 0.79, with a physician's assessment of health) [47,48]. There is limited positive evidence for the reliability (ICC_NPDS _= 0.81, ICC_NPQ _= 0.85, ICC_CNFDS _= 0.84) and responsiveness (r_NPDS _= 0.79, r_NPQ _= 0.81, and r_CNFDS _= 0.65, with a physician's assessment of health on a scale of 0 to 100) of the NPDS, NPQ, and CNFDS [48].

The average time needed to fill out the NDI, NPDS, NPQ, and CNFDS is 8.8, 10.2, 8.4, and 6.8 minutes, respectively [48]. All 4 translated questionnaires show promising results, but we advise using the NDI, because of the excellent methodological quality of the translation process and the good measurement properties of the original version. (Schellingerhout JM, Heymans MW, Verhagen AP, De Vet HC, Koes BW, Terwee CB: Measurement properties of disease-specific questionnaires in patients with neck pain: a systematic review, submitted)

## Discussion

Translated versions of neck-specific questionnaires have been evaluated in 15 different languages. Generally the methodological quality of the translation process is poor, which was mainly due to the fact that the translated version was not pre-tested in the target population. Furthermore, none of the included studies performed a cross-cultural validation. This is necessary to evaluate whether the constructs underlying the original questionnaire are represented adequately by the questionnaire items in the new language. For each questionnaire, except for the Iranian NPDS and Spanish NDI, at least half of the information regarding measurement properties was lacking. Moreover, the evidence for the quality of measurement properties of the translated versions is mostly limited, due to methodological shortcomings of the included studies.

The COSMIN checklist has recently been developed and is based on consensus between experts in the field of health status questionnaires [9]. The COSMIN checklist facilitates a separate judgment of the methodological quality of the included studies and their results. This is in line with the methodology of systematic reviews of clinical trials [15]. The criteria in Table 2 are based on the levels of evidence as previously proposed by the Cochrane Back Review Group [16]. The criteria are originally meant for systematic reviews of clinical trials, but we believe that they are also applicable for reviews on measurement properties of health status questionnaires.

Exclusion of non-English papers may introduce selection bias. However, the leading journals, and as a consequence the most important studies, are published in English. So, research performed in populations with a different native language is generally still published in English. This is illustrated by the large number of articles we retrieved regarding translations of neck-specific questionnaires (see Figure 1). Thus, we argue that the most important translations have been included in our study.

Many studies showed similar methodological shortcomings. Some methodological aspects that need to be improved are: assessment of unidimensionality in internal consistency analysis, the use of stable patients and similar test conditions in studies on reliability and measurement error, and studies on construct validity and responsiveness should be based on predefined hypotheses. We do not discuss these flaws here, because we have elaborated on this subject in a separate paper. (Terwee CB, Schellingerhout JM, Verhagen AP, de Vet HC, Koes BW: Assessing the measurement properties of neck disability questionnaires: room for improvement, submitted)

We pooled the results per language, which neglects the fact that populations might share the same language, but differ in cultural context [3]. However, we think that this did not affect our results, because the only inconsistency in results for the same language version was found for the Chinese NPQ and the populations in the two studies evaluating the Chinese NPQ came from the same region in China and were similar in context [26,27].

A systematic review of the measurement properties of the original version of neck-specific questionnaires showed that for each questionnaire, except for the NDI, at least half of the information regarding measurement properties was lacking. The available results were mainly positive, but the evidence was mostly limited. (Schellingerhout JM, Heymans MW, Verhagen AP, De Vet HC, Koes BW, Terwee CB: Measurement properties of disease-specific questionnaires in patients with neck pain: a systematic review, submitted) This systematic review of translated questionnaires shows similar findings, except that the results for construct validity and responsiveness are more frequently inconsistent or negative. These inconsistencies are in correspondence with those found for translations of the McGill Pain Questionnaire [7]. A possible explanation for this difference in results between original questionnaires and their translated counterparts is the poor methodological quality of the translation process and/or lack of cross-cultural validation [3,4].

A poor translation process and/or lack of cross-cultural validation seem to primarily affect the validity of the questionnaire. This is illustrated by the differences found between the results for structural validity of the translated versions and their original counterparts, and the negative/inconsistent results for hypothesis testing of the translated questionnaires. This is not surprising, as the importance and/or meaning of questionnaire items (e.g. driving, depressed mood) may depend on setting and context. So, a simple translation of the original questionnaire is not sufficient and might affect the underlying constructs. The translation process does not seem to affect the reliability of the questionnaire. This is illustrated by the fact that 95% of the results for internal consistency and reliability are positive, regardless of the methodological quality of the translation process.

A recent review concluded that the translated versions of the NDI into Brazilian-Portuguese, Dutch, French, Korean, and Spanish are of high quality [6]. A possible explanation for discrepancies with our findings is that the methodological quality of the translation process was not taken into account in that review. The same accounts for a state-of-the-art review of the NDI, in which a list of available translations is recommended, without critical appraisal of the quality of the translation process and cross-cultural validation, nor the quality of the measurement properties [5].

This study evaluates the measurement properties of translated versions of neck-specific questionnaires, thereby providing an overview of their availability and making it possible to choose the best questionnaire for a specific study population. However, it is advisable to use them cautiously, since the evidence is mostly limited and for each of these translations, except for the Spanish NDI, at least half of the information regarding measurement properties is lacking. For clinical research and practice we advise to use the following questionnaires: the Catalan, Dutch, English, Iranian, Korean, Spanish and Turkish version of the NDI, the Chinese version of the NPQ, and the Finnish, German and Italian version of the NPDS. This is based on the available results for the measurement properties of these translations, and in the case of the Dutch, English, and Korean NDI on the measurement properties of the original version. (Schellingerhout JM, Heymans MW, Verhagen AP, De Vet HC, Koes BW, Terwee CB: Measurement properties of disease-specific questionnaires in patients with neck pain: a systematic review, submitted) The Greek NDI needs cross-cultural validation and due to poor methodological quality of the available study there is no information on the Swedish NDI. For all other languages it is advisable to first choose the best available original version of the neck-specific questionnaires and perform a high quality translation of this questionnaire. Our previous systematic review on the original versions of all neck-specific questionnaires showed that the NDI was the best questionnaire. (Schellingerhout JM, Heymans MW, Verhagen AP, De Vet HC, Koes BW, Terwee CB: Measurement properties of disease-specific questionnaires in patients with neck pain: a systematic review, submitted)

For future research we recommend performing high quality studies to fill in the information on the unknown measurement properties.

## Conclusion

Translated versions of neck-specific questionnaires have been evaluated in 15 different languages. Generally the methodological quality of the translation process is poor and none of the included studies performed a cross-cultural validation. A substantial amount of information regarding the measurement properties of translated versions of the different neck-specific questionnaires is still lacking or assessed in studies of poor methodological quality. As a result the available evidence on the measurement properties is mostly limited. So, it is advisable to use the available translated questionnaires cautiously. For the time being we advise to use the following questionnaires in clinical research and practice: the Catalan, Dutch, English, Iranian, Korean, Spanish and Turkish version of the NDI, the Chinese version of the NPQ, and the Finnish, German and Italian version of the NPDS. The Greek NDI needs cross-cultural validation and there is no methodologically sound information for the Swedish NDI. Studies of high methodological quality are needed to fill in the unknown measurement properties.

For all other languages we advise to translate the original version of the NDI.

## Competing interests

The authors declare that they have no competing interests.

## Authors' contributions

JMS carried out the bibliographic search, data extraction and assessment of (methodological) quality, and drafted the manuscript. MWH revised the manuscript. APV carried out the bibliographic search and revised the manuscript. HCV was involved in the bibliographic search, data extraction and assessment of (methodological) quality, and revised the manuscript. BWK revised the manuscript. CBT carried out the data extraction and assessment of (methodological) quality, and revised the manuscript. All authors were involved in designing the study. All authors read and approved the final manuscript.

## Pre-publication history

The pre-publication history for this paper can be accessed here:

http://www.biomedcentral.com/1471-2288/11/87/prepub
